# Discovery of a new species of the *Hypoxylon rubiginosum* complex from Iran and antagonistic activities of *Hypoxylon* spp. against the Ash Dieback pathogen, *Hymenoscyphus fraxineus*, in dual culture

**DOI:** 10.3897/mycokeys.66.50946

**Published:** 2020-04-24

**Authors:** Mohammad Javad Pourmoghaddam, Christopher Lambert, Frank Surup, Seyed Akbar Khodaparast, Irmgard Krisai-Greilhuber, Hermann Voglmayr, Marc Stadler

**Affiliations:** 1 Department of Plant Protection, Faculty of Agricultural Sciences, University of Guilan, Rasht, Iran Helmholtz-Zentrum für Infektionsforschung GmbH Braunschweig Germany; 2 Department of Botany and Biodiversity Research, University of Vienna, Rennweg 14, 1030, Wien, Austria University of Guilan Rasht Islamic Republic of Iran; 3 Helmholtz-Zentrum für Infektionsforschung GmbH, Dept. Microbial Drugs, Inhoffenstrasse 7, 38124, Braunschweig, Germany University of Vienna Vienna Austria; 4 Institute of Forest Entomology, Forest Pathology and Forest Protection, Department of Forest and Soil Sciences, BOKU-University of Natural Resources and Life Sciences, Franz-Schwackhöfer-Haus, Peter-Jordan-Straße 82/I, 1190, Vienna, Austria BOKU-University of Natural Resources and Life Sciences Vienna Austria

**Keywords:** Ascomycota, Chemotaxonomy, Chemical ecology, Hypoxylaceae, Natural Products, Taxonomy, one new species

## Abstract

During a survey of xylarialean fungi in Northern Iran, several specimens that showed affinities to the *Hypoxylon rubiginosum* complex were collected and cultured. A comparison of their morphological characters, combined with a chemotaxonomic study based on high performance liquid chromatography, coupled with diode array detection and mass spectrometry (HPLC-DAD/MS) and a multi-locus phylogeny based on ITS, LSU, *rbp2* and *tub2* DNA sequences, revealed a new species here described as *Hypoxylon guilanense*. In addition, *Hypoxylon rubiginosumsensu stricto* was also encountered. Concurrently, an endophytic isolate of the latter species showed strong antagonistic activities against the Ash Dieback pathogen, *Hymenoscyphus fraxineus*, in a dual culture assay in our laboratory. Therefore, we decided to test the new Iranian fungi for antagonistic activities against the pathogen, along with several cultures of other *Hypoxylon* species that are related to *H. rubiginosum*. Our results suggest that the antagonistic effects of *Hypoxylon* spp. against *Hym. fraxineus* are widespread and that they are due to the production of antifungal phomopsidin derivatives in the presence of the pathogen.

## Introduction

*Hypoxylon* Bull., 1791 is one of the largest genera of the Xylariales and comprises more than 200 species, which are mainly associated with angiosperm trees as saprotrophs and endophytes and are predominant in all forest ecosystems of the world (Daranagama et al. 2018; Helaly et al. 2018).

It traditionally belonged to the Xylariaceae until a recent phylogenetic study has resulted in a re-arrangement of the genera of stromatic Xylariales and the resurrection of the family Hypoxylaceae (Wendt et al. 2018). In this study and during the follow-up work by Lambert et al. (2019), genera like *Hypomontagnella* and *Pyrenopolyporus* were segregated from *Hypoxylon*, but the genus remained paraphyletic, indicating that further taxonomic segregation will eventually become necessary.

While the type species of *Hypoxylon*, *H. fragiforme*, belongs to a relatively small clade in the phylogeny of Wendt et al. (2018), the largest clades were comprised of the species of the “*Hypoxylon rubiginosum* complex” sensu Ju and Rogers (1996). Of these species, many had been lumped in *H. rubiginosum*, according to the broad concept established in the first monograph of the genus by Miller (1961). Miller’s concept was mainly based on teleomorph morphology. In their revision of *Hypoxylon*, Ju and Rogers (1996) later recognised that anamorph characters, stromatal pigments and the micromorphology of ascospores and asci (in particular the apical apparatus) constitute valuable diagnostic characters. Modern concepts of the genus combine holomorph morphology with molecular phylogenetic data (Hsieh et al. 2005; Kuhnert et al. 2014). Moreover, secondary metabolite profiles generated by high performance liquid chromatography coupled to diode array detection and mass spectrometry (HPLC-DAD/MS) not only proved highly useful for segregation of species, but even led to the discovery of numerous novel natural products with prominent biological activities (see overviews by Stadler and Hellwig 2005a and Helaly et al. 2018).

*Hypoxylon rubiginosum* and related taxa have been studied rather well on their stromatal secondary metabolites and, in many cases, morphologically similar species may contain entirely different pigments and other compounds (Stadler et al. 2004, 2008; Fournier et al. 2010). Interestingly, several species of the *H. rubiginosum* complex are known to frequently colonise *Fraxinus* species in the temperate Northern hemisphere. In some cases (e.g. *H. cercidicola*, *H. fraxinophilum* and *H. petriniae*), stromata are even almost exclusively found on dead wood of ash trees. They have also been frequently reported as endophytes of the same trees where they produce their stromata (Petrini and Petrini 1985) and are widespread endophytes of other host plants on which their stromata do not even occur (U’Ren et al. 2016). Therefore, the modern concepts of the taxonomy of the Hypoxylaceae take this fact into account and are based on the One Fungus-One Name concept. Some species have even been recognised on the basis of their anamorphic traits (Pažoutová et al. 2013) or their life cycle has been elucidated, based on a polythetic approach, i.e., by comparison of morphological, chemotaxonomic and molecular data of ascospore-derived cultures with endophytic isolates (see Bills et al. 2012 for *H. pulicicidum* and Kuhnert et al. 2014 for *H. griseobrunneum*).

The Ash Dieback disease caused by the introduced apothecial ascomycete *Hym. fraxineus* (Leotiomycetes) has become one of the greatest problems in European forestry and the majority of common ash trees have succumbed to the fungal pathogen. We have recently studied the secondary metabolism of *Hym. fraxineus* (previously also known under the synonyms, *Hym. pseudoalbidus* or *Chalara fraxinea*) and its non-pathogenic domestic relative, *Hym. albidus*, for secondary metabolite production (Halecker et al. 2014, 2018; Surup et al. 2018a). In parallel, we have also isolated endophytic fungi from apparently resistant ash trees in order to find natural antagonists that may be able to combat the devastating disease. One of the best candidates was identified as *H. rubiginosum* and, as reported recently (Halecker et al. 2020), it was found to produce the anti-fungal beta-tubulin inhibitor phomopsidin in dual culture with virulent strains of the pathogen. This compound was first reported from a marine-derived fungus that was originally assigned to the genus *Phomopsis* (Kobayashi et al. 2003). However, it has since then been found in other, terrestrial strains of the same genus, which should now be referred to as *Diaporthe* (Chepkirui and Stadler 2017), a large genus of the order Diaporthales. Interestingly, phomopsidin derivatives have never been reported from cultures of Xylariales before Halecker et al. (2020) found the compound in dual antagonist assays in agar cultures as described above. Moreover, they do not constitute major detectable metabolites of *H. rubiginosum* in the culture media that were used to study the chemotaxonomy of the genus before (cf. Bitzer et al. 2008).

Concurrently, we were about to study the taxonomy of new collections of *Hypoxylon* species originating from Iran that also belong to the *Hypoxylon rubiginosum* complex. Since mycelial cultures of these fungi had just become available, it appeared practical to combine the description of their taxonomy with an evaluation of their antagonistic potential to combat *Hym. fraxineus*. We have also included a number of other *Hypoxylon* species that colonise *Fraxinus* in Europe. The current study therefore provides new evidence on both, the taxonomy and chemical ecology of *Hypoxylon*.

## Materials and methods

### Sample sources

Samples were collected from Guilan and Mazandaran provinces (Northern Iran) during 2015–2017. Parts of corticated branches and trunks bearing Hypoxylaceae stromata were transferred to the laboratory. Details of the specimens used for morphological investigations are listed in the Taxonomy section under the respective descriptions. Specimens have been deposited in the fungarium of the Department of Plant Protection, Faculty of Agricultural Science, University of Guilan, Guilan, Iran (GUM). Living cultures have been deposited in MUCL (Louvain, Belgium).

### Morphological characterisation

Microscopic characters of the teleomorph were observed in distilled water and 10% potassium hydroxide (KOH). Melzer’s reagent was used for staining of the apical ascus apparatus. The numbers of perithecia, ascospores, asci, conidia and conidiophores that were measured for size in the descriptions are 10, 30, 10, 30 and 5, respectively. Specimens were cultured from single ascospore isolates, using 2 % malt extract agar (MEA). For examination of culture macro-morphology, the strains were grown on Difco Oatmeal Agar (OA), following the protocols by Ju and Rogers (1996). Pigment colours were determined as described in the latter monograph, with colour codes following Rayner (1970). Macrophotographs were obtained with a Keyence VHX-6000 microscope. Light microscopy with Nomarski differential interference contrast (DIC) was done using a Zeiss Axio Imager A1 compound microscope, equipped with a Zeiss Axiocam 506 colour digital camera. SEM of ascospores were recorded using a field-emission scanning electron microscope (FE-SEM Merlin, Zeiss, Germany), in a similar fashion as reported previously (Kuhnert et al. 2017).

### DNA extraction, PCR and sequencing

DNA extraction of fresh cultures and amplification of the ITS (nuc rDNA internal transcribed spacer region containing ITS1-5.8S-ITS2), LSU (5' 1200 bp of the large subunit nuc 28S rDNA), *rpb2* (partial second largest subunit of the DNA-directed RNA polymerase II) and *tub2* (partial β-tubulin) loci were performed as described by Wendt et al. (2018). Sequences were generated by an in-house Sanger capillary sequencing solution on campus. Sequences were processed with Geneious 7.1.9 (http://www.geneious.com).

### Molecular phylogenetic analyses

The newly generated sequences were aligned with selected sequences from Wendt et al. (2018) and a combined matrix of the four loci (ITS, LSU, *rpb2* and *tub2*) was concatenated for phylogenetic analyses, with four species (*Biscogniauxia nummularia*, *Graphostroma platystomum*, *Xylaria arbuscula* and *Xylaria hypoxylon*) added as the outgroup. The GenBank accession numbers of sequences are listed in Table 1. Sequences were aligned with the server version of MAFFT (http://mafft.cbrc.jp/alignment/server/, Katoh et al. 2019), checked and refined using BioEdit v. 7.2.6 (Hall 1999). After exclusion of ambiguously aligned regions and long insertions, the final combined data matrix contained 4369 characters, i.e. 578 nucleotides of ITS, 1301 nucleotides of LSU, 1017 nucleotides of *rpb2*, and 1473 nucleotides of *tub2*.

Maximum Parsimony (MP) analyses were performed with PAUP v. 4.0a165 (Swofford 2002). All molecular characters were unordered and given equal weight; analyses were performed with gaps treated as missing data; the COLLAPSE command was set to MINBRLEN. MP analysis of the combined multilocus matrix was done using 1000 replicates of heuristic search with random addition of sequences and subsequent TBR branch swapping (MULTREES option in effect, steepest descent option not in effect). Bootstrap analyses with 1000 replicates were performed in the same way but using 10 rounds of random sequence addition and subsequent branch swapping during each bootstrap replicate.

Maximum Likelihood (ML) analyses were performed with RAxML (Stamatakis 2006) as implemented in raxmlGUI 1.3 (Silvestro and Michalak 2012), using the ML + rapid bootstrap setting and the GTRGAMMA substitution model with 1000 bootstrap replicates. The matrix was partitioned for the different gene regions. In the Results and Discussion, bootstrap values ≤ 70% are considered low, between 70–90% intermediate and ≥ 90% high.

**Table 1.** Isolates and accession numbers of sequences used in the phylogenetic analyses. Type specimens are labelled with HT (holotype) ET (epitype) and PT (paratype). Isolates/sequences in bold were isolated/sequenced in the present study.

Species	Strain number	Origin	Status	GenBank accession numbers				Reference
				ITS	LSU	*rpb2*	*tub2*	
*Annulohypoxylon annulatum*	CBS 140775	Texas	ET	KY610418	KY610418	KY624263	KX376353	Kuhnert et al. (2017), Wendt et al. (2018)
*Annulohypoxylon moriforme*	CBS 123579	Martinique		KX376321	KY610425	KY624289	KX271261	Kuhnert et al. (2017), Wendt et al. (2018)
*Annulohypoxylon truncatum*	CBS 140778	Texas	ET	KY610419	KY610419	KY624277	KX376352	Kuhnert et al. (2017), Wendt et al. (2018)
*Biscogniauxia nummularia*	MUCL 51395	France	ET	KY610382	KY610427	KY624236	KX271241	Wendt et al. (2018)
*Daldinia caldariorum*	MUCL 49211	France		AM749934	KY610433	KY624242	KC977282	Bitzer et al. (2008), Kuhnert et al. (2014), Wendt et al. (2018)
*Daldinia concentrica*	CBS 113277	Germany		AY616683	KY610434	KY624243	KC977274	Triebel et al. (2005), Kuhnert et al. (2014), Wendt et al. (2018)
*Daldinia dennisii*	CBS 114741	Australia	HT	JX658477	KY610435	KY624244	KC977262	Stadler et al. (2014), Kuhnert et al. (2014), Wendt et al. (2018)
*Daldinia petriniae*	MUCL 49214	Austria	ET	AM749937	KY610439	KY624248	KC977261	Bitzer et al. (2008), Kuhnert et al. (2014), Wendt et al. (2018)
*Daldinia placentiformis*	MUCL 47603	Mexico		AM749921	KY610440	KY624249	KC977278	Bitzer et al. (2008), Kuhnert et al. (2014), Wendt et al. (2018)
*Daldinia theissenii*	CBS 113044	Argentina	PT	KY610388	KY610441	KY624251	KX271247	Wendt et al. (2018)
*Daldinia vernicosa*	CBS 119316	Germany	ET	KY610395	KY610442	KY624252	KC977260	Kuhnert et al. (2014), Wendt et al. (2018)
*Entonaema liquescens*	ATCC 46302	USA		KY610389	KY610443	KY624253	KX271248	Wendt et al. (2018)
*Graphostroma platystomum*	CBS 270.87	France		JX658535	DQ836906	KY624296	HG934108	Zhang et al. (2006), Stadler et al. (2014), Koukol et al. (2015), Wendt et al. (2018)
*Hypomontagnella barbarensis*	STMA 14081	Argentina	HT	MK131720	MK131718	MK135891	MK135893	Lambert et al. (2019)
*Hypomontagnella monticulosa*	MUCL 54604	French Guiana	ET	KY610404	KY610487	KY624305	KX271273	Wendt et al. (2018)
*Hypomontagnella submonticulosa*	CBS 115280	France		KC968923	KY610457	KY624226	KC977267	Kuhnert et al. (2014), Wendt et al. (2018)
*Hypoxylon carneum*	MUCL 54177	France		KY610400	KY610480	KY624297	KX271270	Wendt et al. (2018)
*Hypoxylon cercidicola*	CBS 119009	France		KC968908	KY610444	KY624254	KC977263	Kuhnert et al. (2014), Wendt et al. (2018)
*Hypoxylon crocopeplum*	CBS 119004	France		KC968907	KY610445	KY624255	KC977268	Kuhnert et al. (2014), Wendt et al. (2018)
*Hypoxylon fendleri*	MUCL 54792	French Guiana		KF234421	KY610481	KY624298	KF300547	Kuhnert et al. (2014), Wendt et al. (2018)
*Hypoxylon fragiforme*	MUCL 51264	Germany	ET	KC477229	KM186295	KM186296	KX271282	Stadler et al. (2013), Daranagama et al. (2015), Wendt et al. (2018)
*Hypoxylon fuscum*	CBS 113049	France	ET	KY610401	KY610482	KY624299	KX271271	Wendt et al. (2018)
*Hypoxylon griseobrunneum*	CBS 331.73	India	HT	KY610402	KY610483	KY624300	KC977303	Kuhnert et al. (2014), Wendt et al. (2018)
***Hypoxylon guilanense***	**MUCL 57726**	**Iran**	**HT**	**MT214997**	**MT214992**	**MT212235**	**MT212239**	**This study**
*Hypoxylon haematostroma*	MUCL 53301	Martinique	ET	KC968911	KY610484	KY624301	KC977291	Kuhnert et al. (2014), Wendt et al. (2018)
*Hypoxylon howeanum*	MUCL 47599	Germany		AM749928	KY610448	KY624258	KC977277	Bitzer et al. (2008), Kuhnert et al. (2014), Wendt et al. (2018)
*Hypoxylon hypomiltum*	MUCL 51845	Guadeloupe		KY610403	KY610449	KY624302	KX271249	Wendt et al. (2018)
*Hypoxylon investiens*	CBS 118183	Malaysia		KC968925	KY610450	KY624259	KC977270	Kuhnert et al. (2014), Wendt et al. (2018)
*Hypoxylon lateripigmentum*	MUCL 53304	Martinique	HT	KC968933	KY610486	KY624304	KC977290	Kuhnert et al. (2014), Wendt et al. (2018)
*Hypoxylon lenormandii*	CBS 119003	Ecuador		KC968943	KY610452	KY624261	KC977273	Kuhnert et al. (2014), Wendt et al. (2018)
*Hypoxylon musceum*	MUCL 53765	Guadeloupe		KC968926	KY610488	KY624306	KC977280	Bitzer et al. (2008), Kuhnert et al. (2014), Wendt et al. (2018)
*Hypoxylon olivaceopigmentum*	DSM 107924	USA	HT	MK287530	MK287542	MK287555	MK287568	Sir et al. (2019)
*Hypoxylon papillatum*	ATCC 58729	USA	HT	KC968919	KY610454	KY624223	KC977258	Bitzer et al. (2008), Kuhnert et al. (2014), Wendt et al. (2018)
*Hypoxylon perforatum*	CBS 115281	France		KY610391	KY610455	KY624224	KX271250	Wendt et al. (2018)
*Hypoxylon petriniae*	CBS 114746	France	HT	KY610405	KY610491	KY624279	KX271274	Bitzer et al. (2008), Kuhnert et al. (2014), Wendt et al. (2018)
*Hypoxylon pilgerianum*	STMA 13455	Martinique		KY610412	KY610412	KY624308	KY624315	Wendt et al. (2018)
*Hypoxylon porphyreum*	CBS 119022	France		KC968921	KY610456	KY624225	KC977264	Bitzer et al. (2008), Kuhnert et al. (2014), Wendt et al. (2018)
*Hypoxylon pulicicidum*	CBS 122622	Martinique	HT	JX183075	KY610492	KY624280	JX183072	Bills et al. (2012), Wendt et al. (2018)
*Hypoxylon rickii*	MUCL 53309	Martinique	ET	KC968932	KY610416	KY624281	KC977288	Kuhnert et al. (2014), Wendt et al. (2018)
*Hypoxylon rubiginosum*	MUCL 52887	Germany	ET	KC477232	KY610469	KY624266	KY624311	Stadler et al. (2013), Wendt et al. (2018)
***Hypoxylon rubiginosum***	**MUCL 57727**	**Iran**		**MT214998**	**MT214993**	**MT212236**	**MT212240**	**This study**
**Hypoxylon aff. rubiginosum**	**MUCL 57724**	**Iran**		**MT214999**	**MT214994**	**MT212237**	**MT212241**	**This study**
**Hypoxylon aff. rubiginosum**	**MUCL 57725**	**Iran**		**MT215000**	**MT214995**	**MT212238**	**MT212242**	**This study**
*Hypoxylon samuelsii*	MUCL 51843	Guadeloupe	ET	KC968916	KY610466	KY624269	KC977286	Kuhnert et al. (2014), Wendt et al. (2018)
*Hypoxylon texense*	DSM 107933	USA	HT	MK287536	MK287548	MK287561	MK287574	Sir et al. (2019)
*Hypoxylon ticinense*	CBS 115271	France		JQ009317	KY610471	KY624272	AY951757	Hsieh et al. (2005), Wendt et al. (2018)
*Hypoxylon trugodes*	MUCL 54794	Sri Lanka	ET	KF234422	KY610493	KY624282	KF300548	Kuhnert et al. (2014), Wendt et al. (2018)
*Hypoxylon vogesiacum*	CBS 115273	France		KC968920	KY610417	KY624283	KX271275	Kuhnert et al. (2014), Kuhnert et al. (2017), Wendt et al. (2018)
*Jackrogersella cohaerens*	CBS 119126	Germany		KY610396	KY610497	KY624270	KY624314	Wendt et al. (2018)
*Jackrogersella minutella*	CBS 119015	Portugal		KY610381	KY610424	KY624235	KX271240	Kuhnert et al. (2017), Wendt et al. (2018)
*Jackrogersella multiformis*	CBS 119016	Germany	ET	KC477234	KY610473	KY624290	KX271262	Kuhnert et al. (2014), Kuhnert et al. (2017), Wendt et al. (2018)
*Pyrenopolyporus hunteri*	MUCL 52673	Ivory Coast	ET	KY610421	KY610472	KY624309	KU159530	Kuhnert et al. (2017), Wendt et al. (2018)
*Pyrenopolyporus laminosus*	MUCL 53305	Martinique	HT	KC968934	KY610485	KY624303	KC977292	Kuhnert et al. (2014), Wendt et al. (2018)
*Pyrenopolyporus nicaraguensis*	CBS 117739	Burkina_Faso		AM749922	KY610489	KY624307	KC977272	Bitzer et al. (2008), Kuhnert et al. (2014), Wendt et al. (2018)
*Rhopalostroma angolense*	CBS 126414	Ivory Coast		KY610420	KY610459	KY624228	KX271277	Wendt et al. (2018)
*Ruwenzoria pseudoannulata*	MUCL 51394	D. R. Congo	HT	KY610406	KY610494	KY624286	KX271278	Wendt et al. (2018)
*Thamnomyces dendroideus*	CBS 123578	French Guiana	HT	FN428831	KY610467	KY624232	KY624313	Stadler et al. (2010), Wendt et al. (2018)
*Xylaria arbuscula*	CBS 126415	Germany		KY610394	KY610463	KY624287	KX271257	Fournier et al. (2011), Wendt et al. (2018)
*Xylaria hypoxylon*	CBS 122620	Sweden	ET	KY610407	KY610495	KY624231	KX271279	Sir et al. (2016), Wendt et al. (2018)

### HPLC profiling

Stromata of *Hypoxylon* specimens were extracted as described by Kuhnert et al. (2017) and subsequently analysed by high performance liquid chromatography, coupled with diode array and electrospray mass spectrometric detection (HPLC/DAD-ESIMS) instrument settings as described by Halecker et al. (2020). The resulting UV/Vis and mass spectra were compared with an internal database (cf. Bitzer et al. 2008), comprising standards of known Hypoxylaceae.

### Dual culture experiments

Dual cultures of *Hypoxylon* spp. and *Hym. fraxineus* (STMA 18166) were co-incubated on barley-malt agar by inoculation at opposite sites on 9 cm Petri dishes (cf. Halecker et al. 2020) with *Hym. fraxineus* being inoculated one week prior the beginning of the dual culturing due to its slow growth. Axenic cultures, containing only one fungus, were inoculated in parallel as a control group. Growth was documented and observed weekly after incubation in the dark for a maximum of four weeks. Thereafter, the agar plates were extracted with acetone following the method described by Halecker et al. (2020), except that the entire agar plate was extracted instead of the fungal interaction zone.

## Results

### Phylogenetic analyses

**Figure 1.**
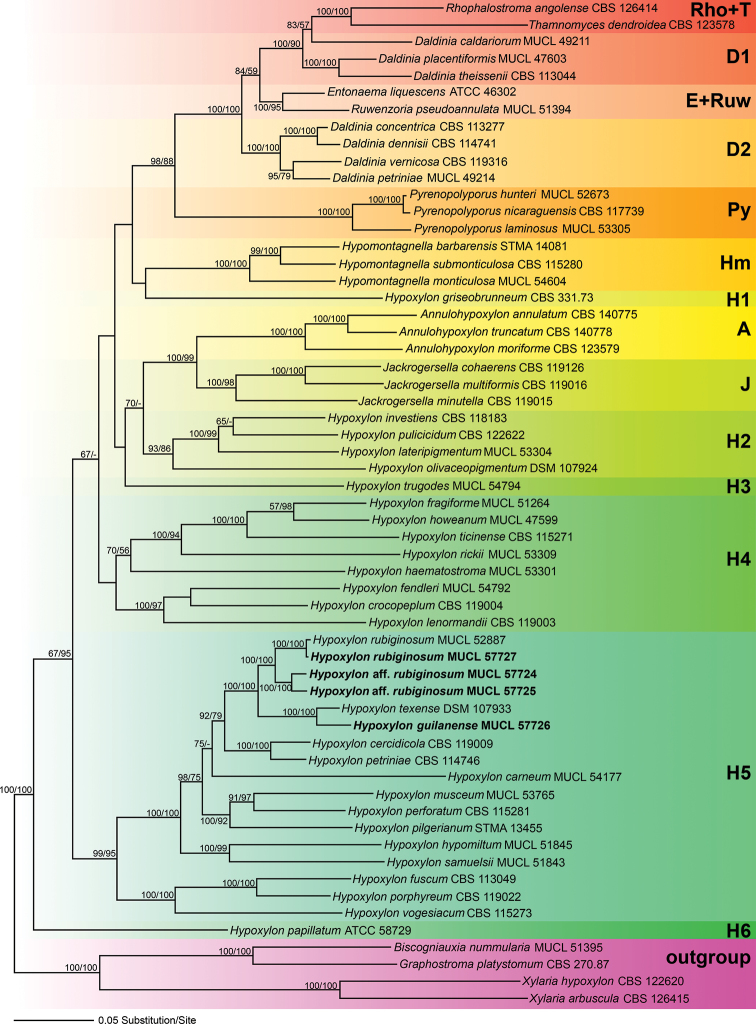
Phylogram of the best ML trees (lnL = −63870.651550) revealed by RAxML from an analysis of the combined ITS–LSU–*rpb2*–*tub2* matrix of selected Xylariales. Strains in bold were sequenced in the current study. ML and MP bootstrap support above 50% are given at the first and second positions, respectively, above or below the branches.

Of the 4369 nucleotide characters of the combined matrix, 1618 are parsimony informative (298 of ITS, 156 of LSU, 487 of *rpb2* and 677 of *tub2*). Fig. 1 shows a phylogram of the best ML tree (lnL = −63870.651550) obtained by RAxML. Maximum parsimony analyses revealed one MP tree comprising 14,014 steps (data not shown). All major groups and deeper, highly supported nodes were consistent between the ML and MP analyses, but topologies of deeper unsupported nodes differed in the MP tree; as these differences are not relevant within the context of our new species, they are not further considered here. The phylogenies reveal a paraphyly of *Hypoxylon*, with the genera *Annulohypoxylon*, *Daldinia*, *Entonaema*, *Jackrogersella*, *Hypomontagnella*, *Pyrenopolyporus*, *Rhopalostroma*, *Ruwenzoria* and *Thamnomyces* embedded within the former. All of the latter genera appeared monophyletic except for *Daldinia* (Fig. 1). All of our new Iranian species and records described below are contained within the highly supported *Hypoxylon* clade **H5**. The new species *H. guilanense* clustered together with *H. texense* with 100% BS support, while sequences of two additional strains (Hypoxylon aff. rubiginosumMUCL 57724) and (Hypoxylon aff. rubiginosumMUCL 57725) formed a highly supported (100% BS support) clade that is the sister group of *H. rubiginosum* (Fig. 1). The sequences of the Iranian collection of *H. rubiginosum* (MUCL 57727) are almost identical to those of the ex-epitype culture (MUCL 52887) and they clustered together with maximum support. As in previous studies, the position of *H. griseobrunneum* and *H. trugodes* could not be resolved within the family. The remaining clades are in accordance with previous results of Wendt et al. (2018).

### Taxonomy

#### Hypoxylon guilanense

Taxon classificationFungiXylarialesXylariaceae

Pourmoghaddam & C. Lambert sp. nov.

5346E2F6-0AB3-5004-8BE0-A23F251504B6

834521

Fig. 2

##### Holotype.

Iran, Guilan Province, Rasht County, Saravan forest, 37°04'26"N, 49°38'13"E, 183 m elev., on fallen branch of *Quercus castaneifolia*, 9 Apr 2015, M.J. Pourmoghaddam. (GUM 989; ex-holotype culture MUCL 57726).

##### Etymology.

Guilanense, refers to its origin in Guilan province, Iran.

##### Teleomorph.

Stromata superficial, hemispherical to pulvinate, up to 2 cm long × 0.1–0.7 cm wide, with conspicuous perithecial mounds, surface Sienna (8), Umber (9) to Buff (45); Scarlet (5) to Orange (7) granules beneath the surface and between the perithecia, with Orange (7) KOH-extractable pigments. Perithecia spherical to obovoid, 0.33–0.66 high × 0.3–0.55 mm wide. Ostioles umbilicate, inconspicuous. Asci not seen. Ascospores smooth, unicellular, brown to dark brown, ellipsoid, inequilateral with narrowly rounded ends, 12–15 × 5–6 µm, with straight germ slit spore-length on convex side; perispore dehiscent in 10% KOH, conspicuous coil-like ornamentation in SEM; epispore smooth.

##### Cultures and anamorph.

Colonies on OA covering a 9 cm Petri dish in 4 wk, at first white, becoming Buff (45), cottony, slightly zonate with diffuse margins; finally, becoming Honey (64). Anamorph not produced in culture.

##### Secondary metabolites.

Orsellinic acid, rubiginosin A and an unknown isomer thereof, as well as mitorubrinol acetate as prevailing stromatal components; cultures produce yet unidentified compounds on barley-malt agar.

##### Notes.

The description of this taxon is based on a single specimen, which shows the salient features of the teleomorph and can be discriminated easily from all previously described species of the *H. rubiginosum* complex. The stromata of the holotype specimen differ from *H. texense* (i.e. the closest relative in the phylogeny), in having stromata with hemispherical to pulvinate shape, Orange (7) KOH-extractable pigments and larger ascospores [12–15 × 5–6 vs. 9.1–10.8 (–11.5) × (4.0–) 4.5–5.4 (–5.7) μm with straight germ slit.

*Hypoxylon guilanense* can also be easily differentiated from *H. rubiginosumsensu stricto* and *H. petriniae* in the peculiar stromatal shape and it also has larger ascospores. *H. cercidicola* differs from *H. guilanense* in having erumpent stromata with discoid shape and smaller ascospores [(9–) 9.5–12 × 5–6 μm)] with straight to slightly sigmoid germ slit. Table 2 compares morphological characters of some other taxa that may be confused with *H. guilanense*.

**Figure 2.**
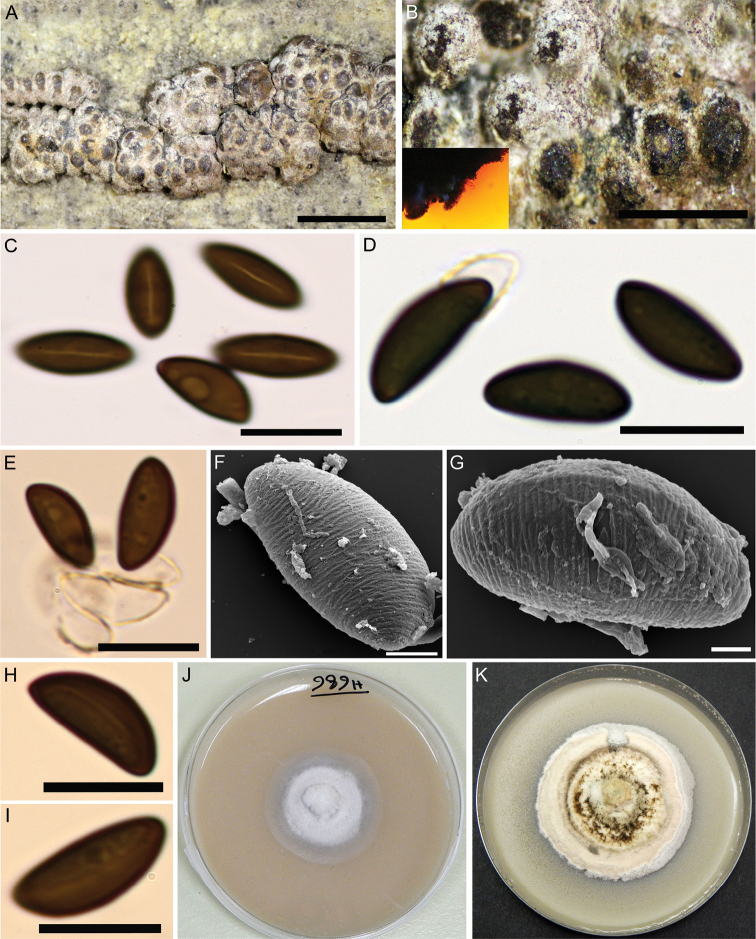
*Hypoxylon guilanense* (Holotype GUM 989) **A** stromatal habit **B** close-up view of stromatal surface, with stromatal pigments in 10% KOH**C, H, I** ascospores in water, with germ-slits **D, E** ascospores in 10% KOH with dehiscent perispore **F, G** ascospore under SEM**J, K** culture on 9 cm OA plates after 1 and 3 wk of incubation (left to right). Scale bars: 2.5 mm (**A**), 1 mm (**B**); 10 µm (**C–E**); 2 µm (**F, G**); 10 µm (**H, I**).

**Table 2.** Diagnostic characters of *Hypoxylon rubiginosumsensu stricto* and closely related species.

Taxon	Stromatal shape	Stromatal surface	KOH-extractable pigments	Ascospores (µm)	Germ slit	Host	Known distribution	Anamorph	Secondary metabolites*
*Hypoxylon canariense*	effused to effused-pulvinate	Fulvous, Dark Brick, Dark Vinaceous	Orange to Sienna	9.5–11.5 × 4.5–5	straight	*Erica*, *Ocotea*, *Laurus*, *Persea*	Spain (Canary Islands)	virgariella-like	Rubiginosins A–C, mitorubrinol acetate
*Hypoxylon carneum*	Effused-pulvinate	Dark purple, Dark vinaceous	Livid violet, absent in old stromata	(7.5–)8–11.5 × 4.5–5	straight	Various angiosperm hosts including *Fraxinus*	probably cosmopolitan but rare	sporothrix-like	Carneic acids A and B, BNT
*Hypoxylon cercidicola*	discoid	Dark brick to Sepia	Orange	(9–)9.5–12 × 5–6	straight to slightly sigmoid	* Fraxinus *	Europe and North America	unknown	Mitorubrin, rubiginosin A and C
*Hypoxylon guilanense*	hemispherical to pulvinate	Sienna, Umber to Buff	Orange	12–15 × 5–6	straight	* Quercus *	Iran	unknown	Rubiginosin A, mitorubrinol acetate
*Hypoxylon lusitanicum*	effused	Brown Vinaceous	Sienna	11–13.5 × 5–7	straight	* Rhamnus *	Portugal	unknown	Rubiginosins A and C, rutilin A
*Hypoxylon petriniae*	irregularly effused	Lilac, Vinaceous to Brown Vinaceous	Orange to Rust	8–11.5(–13) × 4.8–6	straight	*Fraxinus* (mostly); *Acer*, Salicaceae	Western and Central Europe	virgariella-like	Rubiginosin A, BNT
*Hypoxylon retpela*	effused-pulvinate	Livid Vinaceous, Brown Vinaceous,	Orange or Scarlet	(9–)9.5–12 × 4.5–5	straight or slightly sigmoid	unknown	Southeast and East Asia, New Guinea	nodulisporium-like	Mitorubrinol acetate, unknown rubiginosins
*Hypoxylon rubiginosum*	effused-pulvinate	Dark Brick, Brown Vinaceous	Orange	9–13 × 4–5.5	straight	Various angiosperm hosts including *Fraxinus*	Europe, North America	nodulisporium-like	Mitorubrin, rubiginosin A–C, rubiginosic acid, daldinin C
Hypoxylon aff. rubiginosum (GUM 1587)	pulvinate to effused-pulvinate	Luteous, Orange to Ochraceous	Orange	8–10 (–11) × (3–) 4–4.5 (–5)	straight to slightly sigmoid	* Quercus *	Iran	virgariella-like	like *H. rubiginosum*
Hypoxylon aff. rubiginosum (GUM 1588)	pulvinate	Orange to Apricot	Orange	10–15 × 5–6.5	straight to slightly sigmoid	unknown	Iran	not observed-	like *H. rubiginosum*
*Hypoxylon salicicola*	effused	Dark rust to Sepia, Brown Vinaceous	Fulvous to Rust	7.2–9.6 × 3–4.2	straight	*Salix*, rarely on *Fraxinus* and *Prunus*	Northern Europe, USA	nodulisporium-like	Mitorubrinol acetate
*Hypoxylon texense*	effused to effused-pulvinate	Livid Vinaceous to Brown Vinaceous	Rust to Dark Brick	9.1–10.8(–11.5) × (4.0–)4.5–5.4(–5.7)	straight or slightly sigmoid	unknown	USA	nodulisporium to virgariella-like	Rubiginosin A, mitorubrinol acetate, unknown rubiginosins
*Hypoxylon urriesii*	effused	Dark Brick	Orange	11–14.5 × 5–6	straight or slightly sigmoid	unknown	Spain (Canary Islands)	unknown	Mitorubrinol acetate, rubiginosin A

#### Hypoxylon rubiginosum

Taxon classificationFungiXylarialesXylariaceae

(Pers.) Fr., Summa Veg. Scand. II, p. 384. (1849).

8EE961E2-BCEC-52EA-82A7-BCD87578A61F

Fig. 3

##### Teleomorph.

Stromata superficial, effused-pulvinate, up to 8 cm long × 0.3–0.2 cm wide; with inconspicuous to conspicuous perithecial mounds, surface Red (2) to Brick (59); Scarlet (5) to Orange (7) granules beneath the surface and between the perithecia, with Orange (7) to Scarlet (5) KOH-extractable pigments. Perithecia spherical to obovoid, 0.2–0.5 high × 0.15–0.45 mm wide. Ostioles umbilicate, inconspicuous. Asci 8-spored, cylindrical, with amyloid, discoid apical apparatus, 0.5–1 µm high × 1.5–2.5 µm wide, stipe up to 180 µm long and spore-bearing portion 40–80 × 6.5–10 µm. Ascospores smooth, unicellular, brown to dark brown, ellipsoid, inequilateral with narrowly rounded ends, 9–12 (–13) × 4–6 µm, with straight germ slit spore-length on convex side; perispore dehiscent in 10% KOH; epispore smooth.

##### Cultures and anamorph.

Colonies on OA covering a 9 cm Petri dish in 3 wk, at first white, becoming Smoke Grey (105), felty, azonate with diffuse margins; finally becoming Pale Luteous (11) to Straw (46). Asexual morph not produced in culture.

##### Secondary metabolites.

Rubiginosin A and an unknown compound of the mitorubrin / rubiginosin azaphilone family prevalent; cultures produce phomopsidin and unidentified compounds on barley-malt agar.

##### Specimens examined.

Iran, Guilan Province, Siahkal County, Deilaman forest, 36°57'25"N, 49°51'54"E, 1100 m elev., on fallen branch of *Quercus castaneifolia*, 6 Oct 2017 (GUM 1586; culture MUCL 57727); Guilan Province, Shaft County, 36°59'08"N, 49°18'43"E, 594 m elev., on fallen trunk of *Pterocarya fraxinifolia*, 15 Sep 2015 (GUM 1583); Guilan Province, Langaroud County, Liseroud forest, 37°7'44"N, 50°8'41"E, 28 m elev., on fallen branch of *Quercus castaneifolia*, 10 Sep 2016 (GUM 1584); Guilan Province, Talesh County, Gisoum forest, 37°37'30"N, 48°58'15"E, 477 m elev., on fallen branch of *Populus* sp., 20 Oct 2016 (GUM 1585). All specimens collected by M.J. Pourmoghaddam.

##### Notes.

*H. rubiginosumsensu stricto* is a very common fungus in the temperate Northern hemisphere (Stadler et al. 2008) and may occur in subtropical areas, such as Florida, USA (Ju and Rogers 1996). Most of the characters of the Iranian specimens are in accordance with previous descriptions (Stadler et al. 2004), aside from insignificant variations in the size of ascospores.

**Figure 3.**
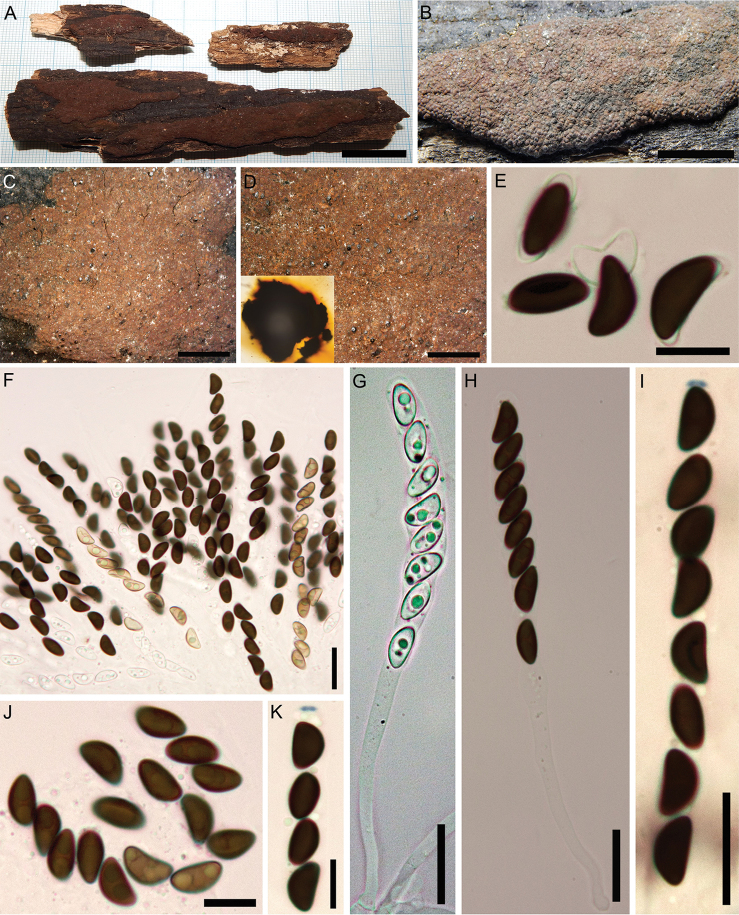
*Hypoxylon rubiginosum* (GUM 1586) **A, B** stromatal habit **C** close-up view of stromatal surface **D** close-up view of stromatal surface, with stromatal pigments in 10% KOH**E** ascospores in 10% KOH with dehiscent perispore **F** mature and immature asci in water **G** immature ascus in water **H** mature ascus in water **I** ascus in Melzer’s reagent **J** ascospores in water **K** ascus tip in Melzer’s reagent. Scale bars: 2 cm (**A**); 1 cm (**B**); 4 mm (**C**); 2 mm (**D**); 10 µm (**E**); 20 µm (**F–I**), 10 µm (**J, K**).

### Additional potentially new species of the H. rubiginosum complex

Below, we describe two collections that may eventually be recognised to represent new species. They appear phylogenetically different from the type specimen, as well as from Iranian records of *H. rubiginosum*, but share salient features with the latter species. It is explained in the Notes why we hesitate to describe them as new taxa in this complicated species complex.

#### Hypoxylon sp. aff. rubiginosum

Taxon classificationFungiXylarialesXylariaceae

GUM 1587

FEA46B16-752F-56D6-9F35-8E509D4414E2

Figs 4 , 5

##### Teleomorph.

Stromata superficial, pulvinate to effused-pulvinate, up to 5 cm long × 0.6–2 cm wide, with inconspicuous to conspicuous perithecial mounds; surface Luteous (12), Orange (7) to Ochreous (44); Orange (7) granules beneath the surface, Orange (7) and Leaden Black (126) granules between the perithecia, with KOH-extractable pigments Orange (7). Perithecia obovoid, compressed-obovoid to spherical, 0.27–0.50 high × 0.23–0.35 mm wide. Ostioles umbilicate, inconspicuous, usually overlain with conspicuous white substance. Asci 8-spored, cylindrical, with amyloid, discoid apical apparatus, 0.5–1 µm high × 2–2.5 µm wide, stipe up to 180 µm long; spore-bearing portion 80–100 × 5.5–7 µm. Ascospores smooth, unicellular, brown to dark brown, ellipsoid, inequilateral with narrowly rounded ends, 8–10 (–11) × (3–) 4–4.5 (–5) µm, with straight to slightly sigmoid germ slit spore-length on convex side; perispore dehiscent in 10% KOH, conspicuous coil-like ornamentation in SEM; epispore smooth.

**Figure 4.**
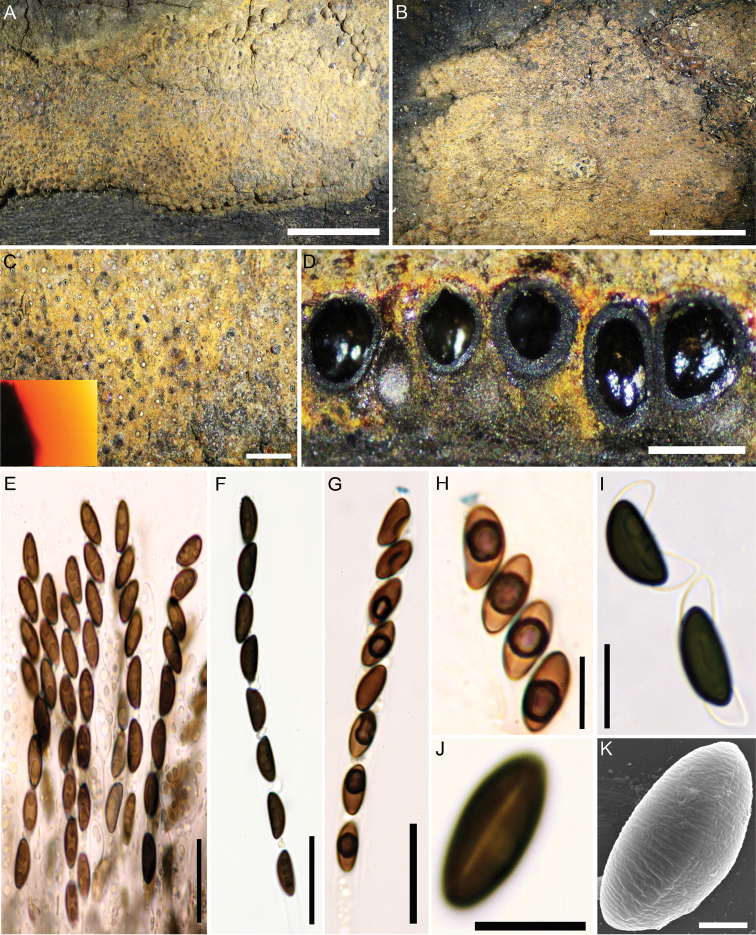
Hypoxylon aff. rubiginosum (GUM 1587) **A, B** stromatal habit **C** close-up view of stromatal surface, with stromatal pigments in 10% KOH**D** stroma in section showing perithecia and ostioles **E** mature and immature asci in water **F** ascus in water **G** ascus in Melzer’s reagent **H** ascus tip in Melzer’s reagent **I** ascospores in 10% KOH with dehiscent perispore **J** ascospore in water, with germ-slit **K** ascospore under SEM. Scale bars: 5 mm (**A, B**); 1 mm (**C**); 0.5 mm (**D**); 20 µm (**E–G**); 10 µm (**H–J**); 2 µm (**K**).

##### Cultures and anamorph.

**Figure 5.**
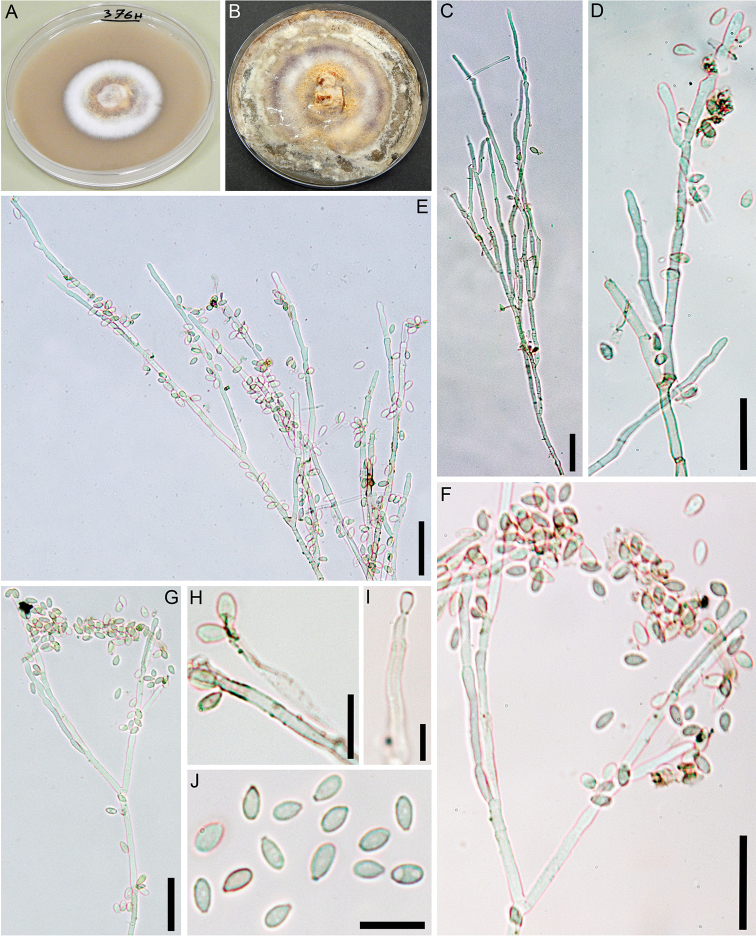
Culture and anamorphic structures of Hypoxylon aff. rubiginosum (GUM 1587) on OA**A, B** surface of colony after 1 and 8 wk of incubation (respectively, left to right) **C–G** general view of anamorph structure with virgariella-like branching patterns **H, I** conidiogenous cells and immature conidia **J** mature conidia. Scale bars: 20 µm (**C–G**); 10 µm (**H–J**).

Colonies on OA covering a 9 cm Petri dish in 3 wk, at first white, becoming Luteous (12) from outwards, cottony, slightly zonate with diffuse margins; finally, attaining a variety of different colours. Conidiogenous structure branching virgariella-like as defined by Ju and Rogers (1996), (Fig. 5C–G). Conidiophores hyaline, smooth to finely roughened. Conidiogenous cells hyaline, smooth to finely roughened, 15–30 × 2–3 μm. Conidia hyaline, smooth to ellipsoid, 4–6 × 2–3 μm.

##### Specimen examined.

Iran, Guilan Province, Astaneh-Ashrafieh County, Safra-Basteh forest, 37°20'19"N, 49°58'26"E, 14 m elev., on fallen branch of *Quercus castaneifolia*, 4 Oct 2016, M.J. Pourmoghaddam (GUM 1587; culture MUCL 57724).

##### Notes.

This specimen resembles *H. rubiginosum* in many respects. However, it has slightly smaller ascospores [8–10 (–11) × (3–) 4–4.5 (–5) vs. 9–13 × 4–5.5 µm] and the germ slit of the ascospores is often slightly sigmoid. The most significant differences were noted in the anamorphic structures with virgariella-like branching patterns. This anamorph actually resembles that of *H. petriniae*. However, this species is normally associated with *Fraxinus* and differs from Hypoxylon aff. rubiginosumGUM 1587 in having Lilac (54), Vinaceous (57) to Brown Vinaceous (84) stromatal surface colours (owing to the presence of BNT, which was not found in the Iranian specimen). It also differs in having more elongate to irregularly effused stromata with black margins and its ascospores are larger (8–11.5 (–13) × 4.8–6 μm) and have a straight germ slit.

#### Hypoxylon sp. aff. rubiginosum

Taxon classificationFungiXylarialesXylariaceae

GUM 1588

69BA22CB-5B18-534E-91A0-A4637BC8E989

Fig. 6

##### Teleomorph.

Stromata superficial, pulvinate, up to 1 cm long × 0.2–0.5 cm wide, with inconspicuous to conspicuous perithecial mounds; surface Orange (7) to Apricot (42); Orange (7) granules beneath the surface and Laeden Black (126) granules between the perithecia, with Orange (7) KOH-extractable pigments. Perithecia obovoid to compressed-obovoid, 0.35–0.65 high × 0.3–0.45 mm wide. Ostioles umbilicate, inconspicuous. Asci with amyloid, discoid apical apparatus, 1–1.5 µm high × 2–3 µm wide, stipe up to 160 µm and spore-bearing portion 70–100 × 6–8 µm long. Ascospores smooth, unicellular, brown to dark brown, ellipsoid, inequilateral with narrowly-rounded ends, 10–15 × 5–6.5 µm, with straight to slightly sigmoid germ slit spore-length on convex side; perispore dehiscent in 10% KOH; epispore smooth.

##### Cultures and anamorph.

Colonies on OA covering a 9 cm Petri dish in 3 wk, at first white, becoming whitish, cottony, azonate with entire margins; remaining mainly uncoloured with Pale Luteous tinges. Anamorph not produced in culture.

##### Specimen examined.

Iran, Mazandaran Province, Tonekabon County, Do-hezar forest, 36°42'30"N, 50°49'43"E, 456 m elev., on dead branches (host unknown), 28 Oct 2016, M.J. Pourmoghaddam (GUM 1588; culture MUCL 57725).

##### Notes.

This specimen is morphologically similar to Hypoxylon aff. rubiginosumGUM 1587, but it can be distinguished by its larger ascospores [10–15 × 5–6.5 vs. 8–10 (–11) × (3–) 4–4.5 (–5) μm]. *H. rubiginosumsensu stricto* differs from this specimen in having smaller ascospores [(8–) 9–12 × 4–5.5 vs. 10–15 × 5–6.5 μm]. In addition, the stromatal secondary metabolite profile is similar to that of *H. rubiginosum* with two unknown azaphilone compounds of the mitorubrin / rubiginosin family (**UC 2**, retention time = 8.7 min, 442 Dalton and **UC 3**, RT = 10.6 min, 884 Da) and rubiginosin A. *H. guilanense* differs from Hypoxylon aff. rubiginosumGUM 1588 in having stromata with hemispherical to pulvinate shape and difference in average ascospores sizes (12–15 × 5–6 vs. 10–15 × 5–6.5 μm) with straight germ slit. *H. texense* differs from Hypoxylon aff. rubiginosumGUM 1588 in having Rust (39) to Dark Brick (86) KOH-extractable pigments and much smaller ascospores [9.1–10.8 (–11.5) × (4.0–) 4.5–5.4 (–5.7) vs. 10–15 × 5–6.5 μm].

**Figure 6.**
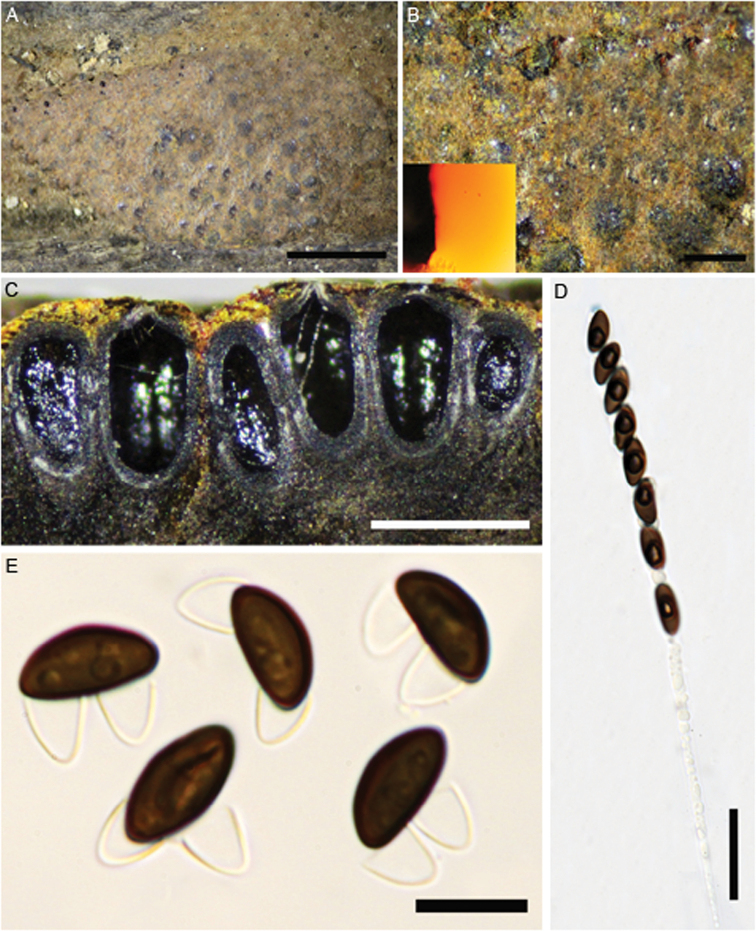
Hypoxylon aff. rubiginosum (GUM 1588) **A** stromatal habit **B** close-up view of stromatal surface, with stromatal pigments in 10% KOH**C** section of stroma showing perithecia and ostioles **D** ascus in Melzer’s reagent **E** ascospores in 10% KOH with dehiscent perispore. Scale bars: 2.5 mm (**A**); 0.5 mm (**B, C**); 20 µm (**D**); 10 µm (**E**).

### HPLC profiling of stromata

Amongst the four studied Iranian *Hypoxylon* spp., five major metabolites could be identified. Beneath common secondary metabolites of the *H. rubiginosum* complex like orsellinic acid (**1**, Stadler et al. 2008), mitorubrin acetate (**2**, Steglich et al. 1974; Stadler et al. 2001) and rubiginosin A (**3**, Quang et al. 2004), three more non-assignable compounds were detected. UV/Vis data of these metabolites tentatively suggested affinities to the rubiginosin azaphilone family (Fig. 9, **UC 2** and **3**) with one unknown compound sharing the same mass and UV/Vis maxima of mitorubrinol (**4**), which could possibly constitute a yet undescribed isomer (**URg**). Compounds **URg** and **UC 2** have been reported from *H. texense*, which was recently discovered in Texas, USA, as another species of the *H. rubiginosum* complex (Sir et al. 2019). These findings are further reflected in the taxonomic part of this paper.

### HPLC profiling of extracts from single and dual culture experiments (Figs 10, 11)

**Figure 7.**
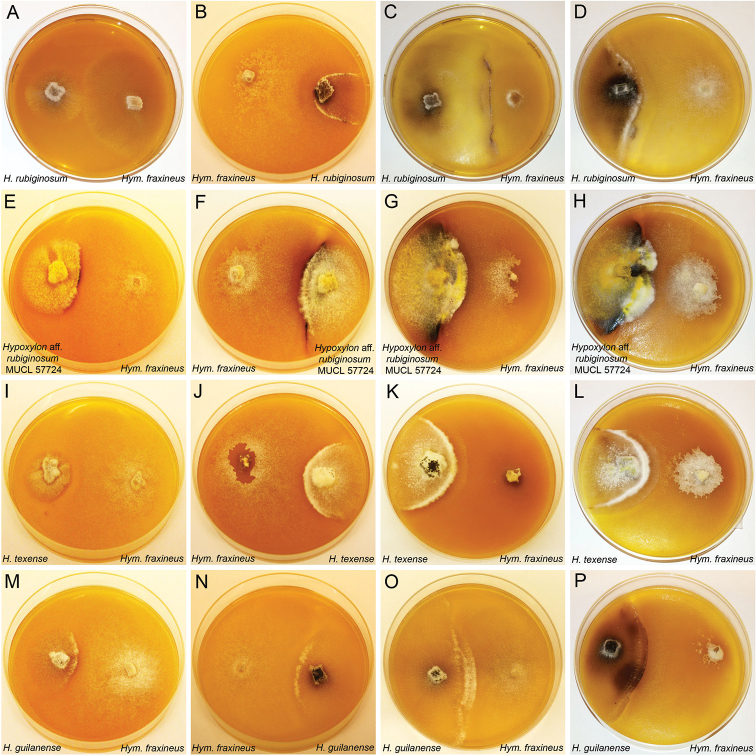
Illustration of antagonist test by dual culture technique of *Hypoxlon* spp. and *Hymenoscyphus fraxineus* on barley-malt agar in 9-cm diam. plates **A** dual culture of *H. rubiginosum* (MUCL 47152) against *Hym. fraxineus* (STMA 18166) after 1 wk of incubation **B** dual culture of *H. rubiginosum* (MUCL 47152) against *Hym. fraxineus* (STMA 18166) after 2 wk of incubation **C** dual culture of *H. rubiginosum* (MUCL 47152) against *Hym. fraxineus* (STMA 18166) after 3 wk of incubation **D** dual culture of *H. rubiginosum* (MUCL 47152) against *Hym. fraxineus* (STMA 18166) after 4 wk of incubation **E–H** (Hypoxylon aff. rubiginosumMUCL 57724) against *Hym. fraxineus* after 1, 2, 3, 4 wk **I–L***H. texense* (DSM 107933) against *Hym. fraxineus* after 1, 2, 3, 4 wk **M–P***H. guilanense* (MUCL 57726) against *Hym. fraxineus* after 1, 2, 3, 4 wk.

**Figure 8.**
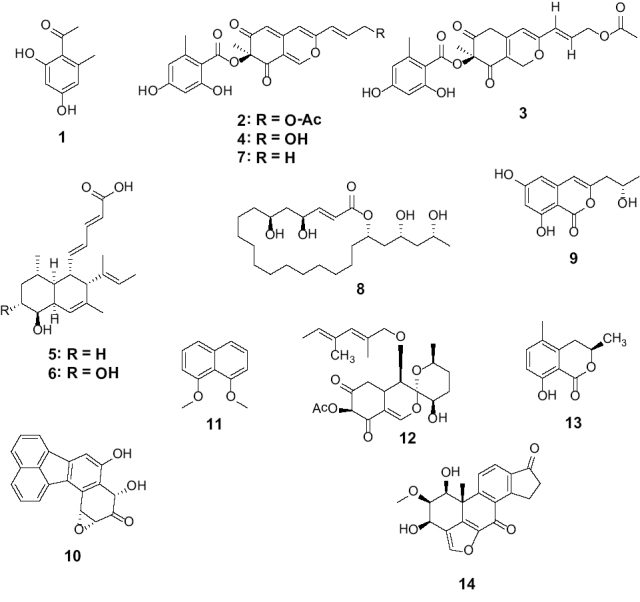
Chemical structures of discussed secondary metabolites. Orsellinic acid (**1**); mitorubrinol acetate (**2**); rubiginosin A (**3**); mitorubrinol (**4**); phomopsidin (**5**); 10-hydroxyphomopsidin (**6**); mitorubrin (**7**); rickiol A (**8**); orthosporin (**9**); daldinone B (**10**); 1,8-dimethoxynaphthalene (**11**); daldinin F (**12**); 5-methyl mellein (**13**); viridiol (**14**).

In total, 32 different *Hypoxylon* strains were screened for production of phomopsidin (**5**, Kobayashi et al. 2003) and 10-hydroxyphomopsidin (**6**, Halecker et al. 2020). Due to the availability of well-studied strains of *H. rubiginosum*, *H. perforatum* and *H. petriniae* in public culture collections, a pre-screening was conducted to confirm production of **5** and **6** (with 13, 7 and 4 strains each, respectively (cf. Table 3, Fig. 11H). Out of these 24 strains, 16 emerged as producers of compound **5** and partially **6** (12 strains). Compound **6** was not detected in the absence of **5**. Out of those, two strains of *H. rubiginosum* (MUCL 47152 and MUCL 47970), one representative of *H. perforatum* (MUCL 47187) and one culture of *H. petriniae* (MUCL 53756) were selected for further testing against *Hym. fraxineus*. The results are illustrated, based on four examples in Fig. 7, showing the dual cultures after 1–4 weeks of incubation. The chemical structures are shown in Fig. 8 and selected chromatographic data are depicted in Figs 10, 11.

**Table 3.** Identified secondary metabolites in axenic cultures on barley-malt medium of the surveyed strains. Strains in **bold** have been used concurrently against STMA 18166 (*Hymenoscyphus fraxineus*) in an antagonism assay. Identified compounds: **5**: phomopsidin; **6**: 10-hydroxyphomopsidin; **8**: rickiol A; **9**: orthosporin **10**: daldinone B; **11**: 1,8-dimethoxynaphtahlene; **13**: 5-methyl-mellein. Identified stromal azaphilone groups detected in culture: MI = Mitorubrin type; NA = Naphthalene type; DA =Daldinin type. For chemical structures, see Fig. 8.

Organism	Strain	Culture metabolites			Stromal metabolites		
		5	6	Others	MI	NA	DA
***Hypoxylon guilanense***	**MUCL 57726**	–	–	–	–	–	–
**Hypoxylon aff. rubiginosum**	**MUCL 57724**	+	+	–	+	–	–
*Hypoxylon rubiginosum*	MUCL 57727	+	–	–	–	–	–
Hypoxylon aff. rubiginosum	MUCL 57725	+	+	–	–	–	–
*Hypoxylon perforatum*	MUCL 57728	–	–	**10**	–	–	–
*Hypoxylon perforatum*	CBS 119011	–	–	**10**	–	–	–
*Hypoxylon perforatum*	MUCL 47187	+	+	–	–	–	–
*Hypoxylon perforatum*	MUCL 54798	–	–	**10**	–	–	–
*Hypoxylon perforatum*	STMA 13041	+	+	–	–	–	–
*Hypoxylon perforatum*	STMA 14051	–	–	**10**	–	–	–
*Hypoxylon perforatum*	CBS 140779	–	–	**10**	–	–	–
*Hypoxylon petriniae*	MUCL 53756	+	+	–	–	–	–
*Hypoxylon petriniae*	STMA 12020	–	–	–	–	–	–
*Hypoxylon petriniae*	STMA 13303	–	–	–	–	–	–
*Hypoxylon petriniae*	STMA 13313	–	–	**10**	–	–	–
*Hypoxylon rubiginosum*	MUCL 2354	–	–	–	–	–	–
***Hypoxylon rubiginosum***	**MUCL 47152**	+	+	**9, 10**	–	+	–
***Hypoxylon rubiginosum***	**MUCL 47970**	+	+	**9, 10**	–	+	–
*Hypoxylon rubiginosum*	MUCL 47150	+	–	–	+	–	–
*Hypoxylon rubiginosum*	MUCL 52672	+	+	–	+	–	–
*Hypoxylon rubiginosum*	MUCL 54624	–	–	**8**	–	–	–
*Hypoxylon rubiginosum*	MUCL 2709	–	–	–	–	–	–
*Hypoxylon rubiginosum*	MUCL 34183	+	+	**13**	–	–	–
*Hypoxylon rubiginosum*	MUCL 47147	+	–	–	+	–	–
*Hypoxylon rubiginosum*	STMA 04040	+	+	–	+	–	–
*Hypoxylon rubiginosum*	STMA 07027	+	+	–	–	–	–
*Hypoxylon rubiginosum*	STMA 13346	+	+	–	–	–	–
*Hypoxylon rubiginosum*	STMA 17058	+	+	–	–	–	–
***Hypoxylon cercidicola***	**MUCL 54180**	+	–	**13**	–	–	–
***Hypoxylon fuscum***	**STMA 13090**	–	–	**11, 13**	–	+	+
***Hypoxylon texense***	**DSM 107933**	–	–	–	+	–	–
***Hypoxylon crocopeplum***	**CBS 119004**	–	–	–	+	–	–
***Hypoxylon carneum***	**MUCL 54177**	–	–	**10**	–	–	–

**Figure 9.**
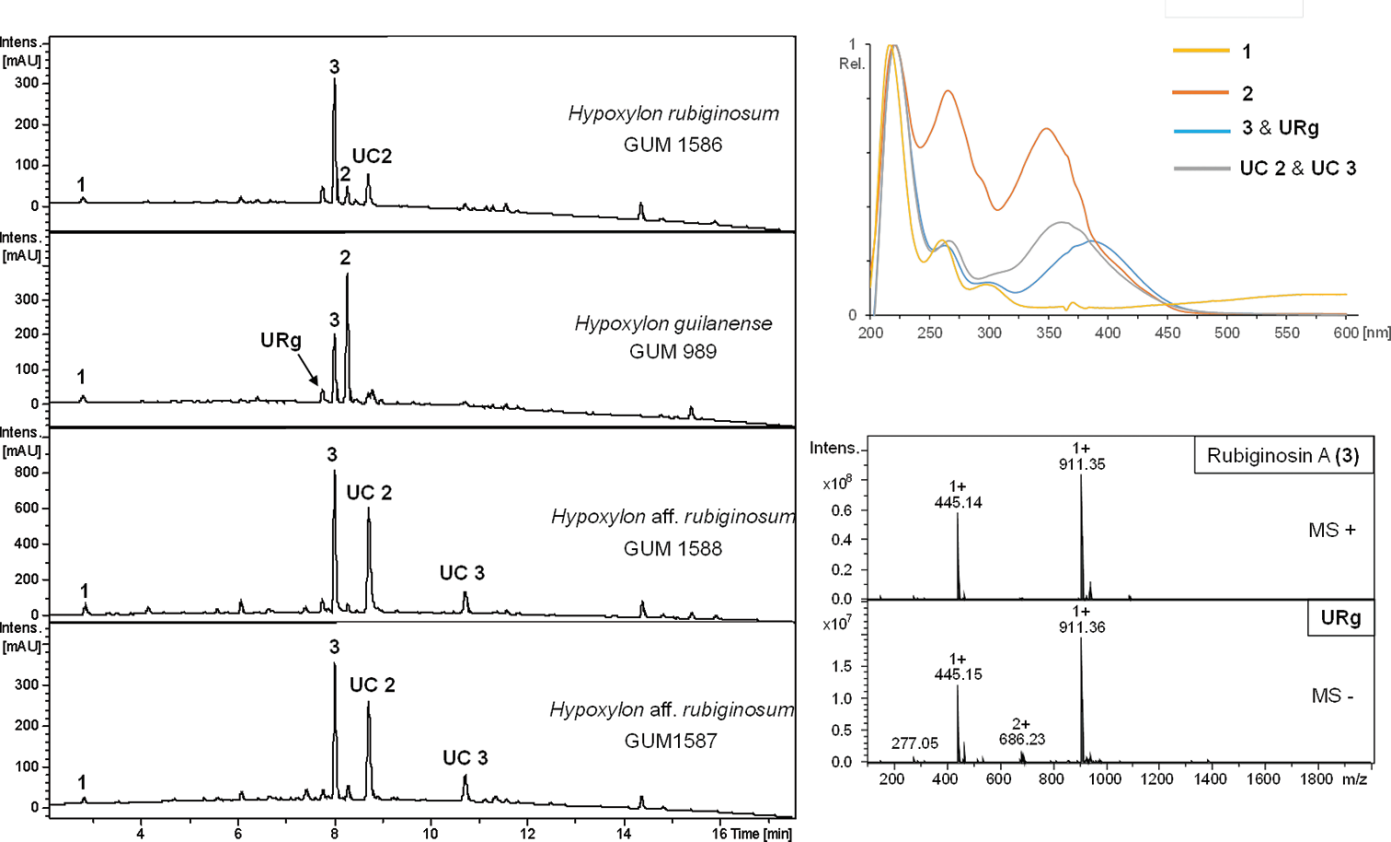
HPLC-UV profiles at 210 nm derived from stromal extracts of strains *H. rubiginosum* (GUM 1586), *H. guilanense* (from holotype) and Hypoxylon aff. rubiginosumGUM 1587 and GUM 1588. UV/Vis spectra are shown for orsellinic acid (**1**), mitorubrinol acetate (**2**), rubiginosin A (**3**), an unknown rubiginosin A – like derivative (**URg**) and rubiginosin – like derivatives (**UC 2** and **UC 3**). ESI mass spectra are shown for compounds **URg** and **2**.

**Figure 10.**
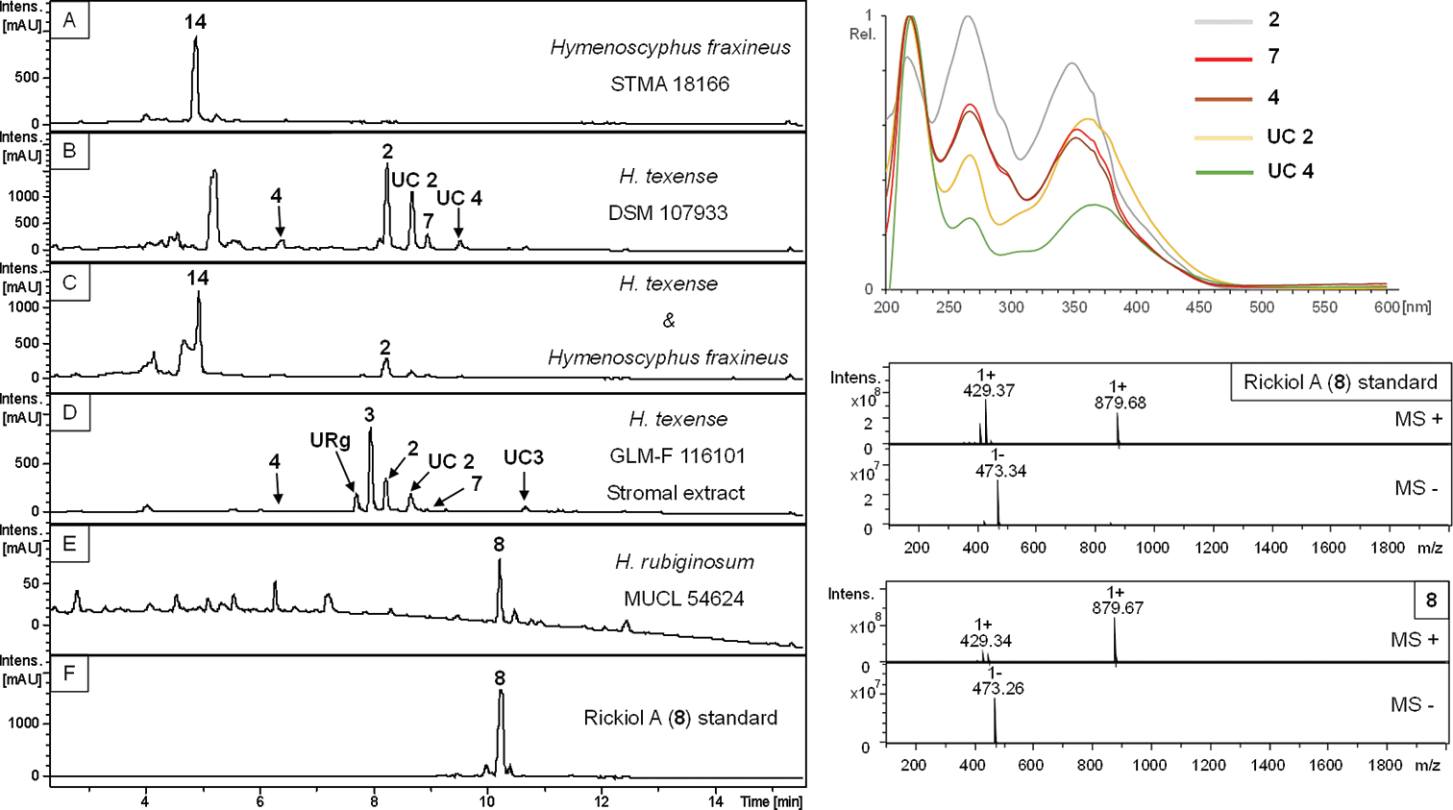
HPLC-UV profiles at 210 nm derived from barley-malt agar (**A–C, E**) and stromal (**E**) extracts and compound standard (**F**). UV/Vis spectra are shown for identified compounds in mono- and dual culture (**C**) experiments of STMA 18166 (*Hym. fraxineus*, **A**) and DSM 107933 (*H. texense*, **B**; **UC 2, 4** – unknown compounds); stromal metabolites (**4** – mitorubrinol; **URg** – unknown rubiginosin A derivative; **3** – rubiginosin A; **2** – mitorubrinol acetate; **7** – mitorubrin; **UC2** – Unknown compound 2 of GLM-F116101 (*H. texense*, **D**), and ... ESI mass spectra of 8 in positive and negative modes... of 8 **8** (rickiol A, **F**) identified in the mono culture extract of MUCL 54624 (*H. rubiginosum*, **E**).

**Figure 11.**
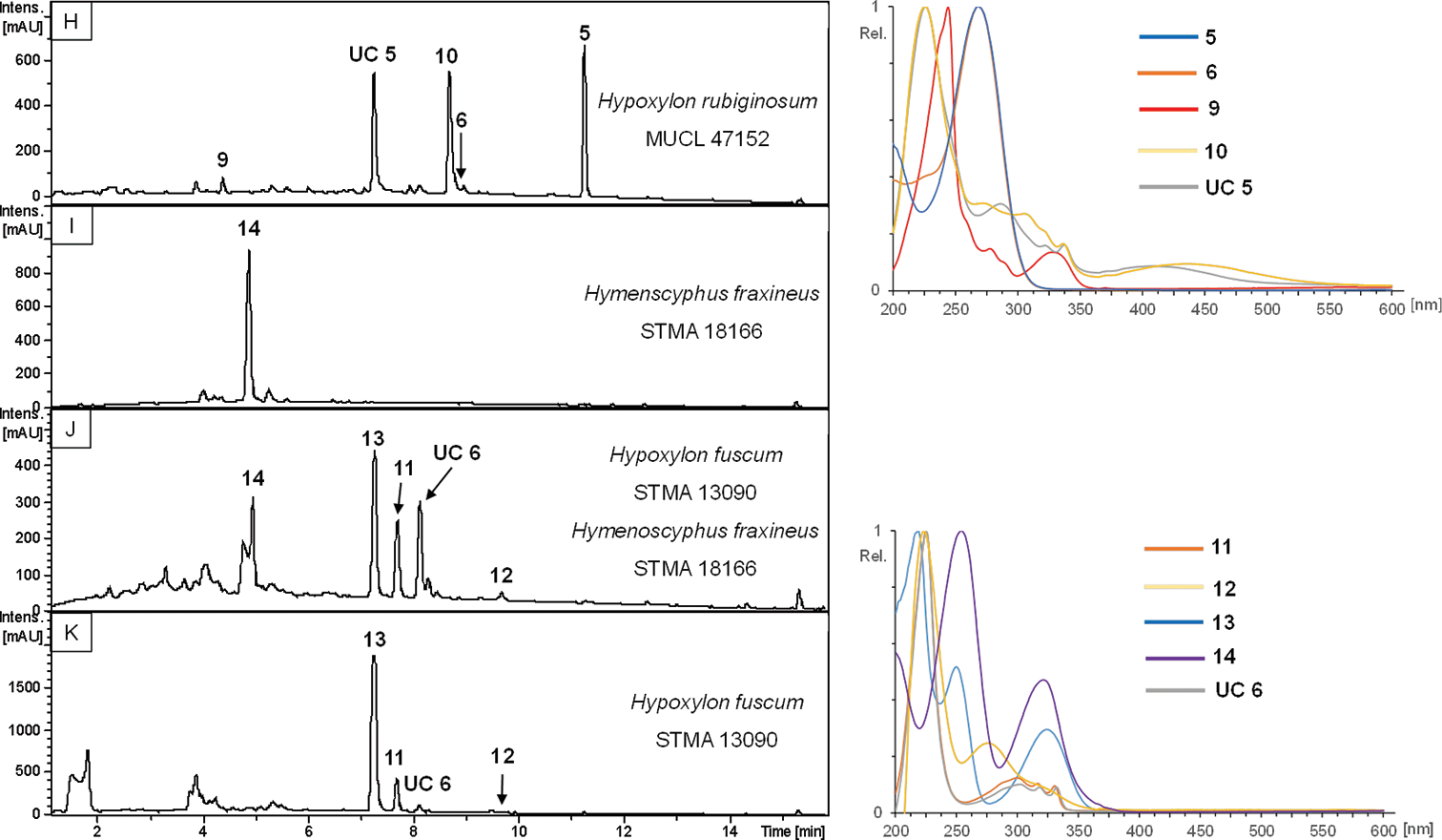
HPLC-UV chromatograms at 210 nm from mono cultural barley-malt agar extracts of MUCL 47152 (*H. rubiginosum*), STMA 18166 (*Hym. fraxineus*), STMA 13090 (*H. fuscum*) and one dual culture experiment thereof. UV/Vis spectra are shown for phomopsidin (**5**), 10-hydroxyphomopsidin (**6**), orthosporin (**9**), daldinone B (**10**), 1,8-dimethoxynaphthalene (**11**), daldinin F (**12**), 5–methylmellein (**13**), viridiol (**14**) and an unidentifiable compound (**UC 6**) after comparison of data with internal databases. The UV signal of **UC 6** was enhanced in the dual culture extract.

Strikingly, during evaluation and comparison of the HPLC UV/Vis chromatograms with our internal database, the mitorubrin derivatives **2, 4** and **7** were identified by direct comparison of chromatograms derived from extracts of stromata and cultures of the ex-type strain and the holotype of *H. texense* (Sir et al. 2019; Figs 7 I–L, 11B, D). Beneath the aforementioned **UC 2**, another yet undescribed compound was revealed (**UC 4**). The main metabolite of the mono cultural extract of MUCL 54624 was identified by comparison of UV/Vis and MS data as rickiol A (**8**; Fig. 11E–F), previously described from *H. rickii* (Surup et al. 2018b). Orthosporin (**9**; Quang et al. 2002), daldinone B (**10**; Stadler et al. 2008) was identified by comparison with an internal database in several strains of *H. rubiginosum*, *H. perforatum* and *H. petriniae* (cf. Tables 3, 4, Fig. 11H). The mono cultural extract of *H. fuscum* (STMA 13090) revealed 1,8 dimethoxynaphthalene (**11**; Chang et al. 2014) and another unidentified peak (**UC 6**, Fig. 11J, K) with an identical UV/Vis spectrum as **11**, as well as traces of Daldinin F (**12**; Quang et al. 2004) and 5- methylmellein (**13**; Stadler et al. 2005b) as the main product. Interestingly, the UV signal of **UC 7** was visibly enhanced in the chromatogram derived from the dual culture extract. The phytotoxic compound viridiol (**14**; Figs 10A, 11I–K) was found in both mono and dual culture extracts of *Hym. fraxineus* (Andersson et al. 2010; Halecker et al. 2020).

**Table 4.** Identified secondary metabolites in dual culture (barley-malt medium with *Hymenoscyphus fraxineus*) of the surveyed strains listed in Table 3. Identified compounds: **5**: phomopsidin; **6**: 10-hydroxyphomopsidin; **8**: rickiol A; **9**: orthosporin; **10**: daldinone B; **11**: 1,8-dimethoxynaphtahlene; **13**: 5-methyl-mellein. Identified stromal azaphilone groups detected in culture: **MI** = Mitorubrin type; **NA** = Naphthalene type; **DA** = Daldinin type. For chemical structures, see Fig. 8.

Organism	Strain	Culture metabolites			Stromal metabolites	
		5	6	Others	MI	NA
*Hypoxylon cercidicola*	MUCL 54180	+	–	**13**	–	–
*Hypoxylon fuscum*	STMA 13090	–	–	**11, 13**	–	+
*Hypoxylon texense*	DSM 107933	–	–	–	+	–
*Hypoxylon crocopeplum*	CBS 119004	–	–	–	+	–
*Hypoxylon perforatum*	MUCL 47187	+	–	–	+	–
*Hypoxylon petriniae*	MUCL 53756	+	–	–	–	–
Hypoxylon aff. rubiginosum	MUCL 57724	+	+	–	+	–
*Hypoxylon rubiginosum*	MUCL 47152	+	–	**9, 10**	–	+
*Hypoxylon rubiginosum*	MUCL 47970	+	–	**9, 10**	–	+
*Hypoxylon guilanense*	MUCL 57726	–	–	–	–	–
*Hypoxylon carneum*	MUCL 54177	–	–	**10**	–	–

## Discussion

The present study dealt with the identification of *Hypoxylon* species from Northern Iran based on morphological, chemotaxonomic and phylogenetic data, focusing on the *H. rubiginosum* complex. The specimens encountered appeared morphologically and chemotaxonomically related to *H. rubiginosumsensu stricto*, as revealed from their morphology and secondary metabolite profiles. While the majority of specimens were assigned to typical *H. rubiginosum*, we have encountered a new taxon that significantly deviates from the complex in both stromatal and ascospore morphology and appears most closely related to a species that was so far only reported from the southern USA (Sir et al. 2019). Furthermore, we found two specimens that slightly differed in one or two characters from typical *H. rubiginosum* and also showed deviating positions in the phylogenetic trees, but are so far only known from single collections. Attempts should be made to encounter additional specimens of these fungi, which may eventually lead to their recognition as new species. The recent study on intragenomic polymorphisms in Hypoxylaceae has suggested that molecular data alone may be misleading in this family and new taxa should be based on multiple records sharing the same genotypic and phenotypic features (Stadler et al. 2020). Hsieh et al. (2005) have already established that protein-coding genes provide a better resolution in the Hypoxylaceae than ITS and finally even omitted this locus from the phylogeny and rather decided to focus on *tub2* and alpha-actin sequences. Kuhnert et al. (2014) also found *tub2* to be more suitable than ITS in their phylogeny, based on material from the Caribbean.

Our phylogenetic analyses confirmed previous results (Wendt et al. 2018; Lambert et al. 2019; Sir et al. 2019), suggesting that the genus *Hypoxylon* appears paraphyletic in Hypoxylaceae, with a relatively small clade comprising the type species *H. fragiforme* as “core group” to which members of the *Hypoxylon rubiginosum* complex form a sister clade. The genus will eventually need to be further subdivided, but molecular data for the majority of known species remain incomplete and such a task should only commence as the phylogenetic data matrix has increased. Our study further contributed to this monumental task by adding some data on representatives from the Middle East, a geographic area that has certainly not been as well explored as Western Europe and other parts of the world.

A main objective of this work was to assess the antagonistic potential of the newly isolated cultures and some strains of related species against an important pathogen, following the recent discovery that an endophytic isolate of *H. rubiginosum* from a resistant ash tree inhibited the growth of the alien pathogen, *Hym. fraxineus* (Halecker et al. 2020). Assessment of axenic cultures of the *Hypoxylon* species in a single medium (barley-malt) led to the detection of phomopsidin in one out of five strains of *H. petriniae*, two out of seven strains of *H. perforatum* and ten out of 13 strains of *H. rubiginosum*. The stromata of these three taxa have been frequently reported from *Fraxinus* and it is plausible that they all occur as endophytes in this host and only form the stromata on dead host tissues. On the other hand, phomopsidin was not detected in other related, but apparently rare species like *H. texense*, *H. crocopeplum* and *H. carneum*. Only the two latter species, however, were represented in our study by cultures that were isolated from stromata growing on *Fraxinus* wood. In addition, our results need to be further validated because we cannot exclude that some of the strains, which have been kept in culture collections for many years, may have degenerated. In any case, our results suggest that phomopsidin is not a specific marker for the species complex or for *H. rubiginosumsensu stricto.* As the compound is preferentially observed in dual cultures, its biosynthesis may be under control of epigenetic effectors. Therefore, in the future, it would be useful to evaluate a broader range of ascospore-derived cultures of *Hypoxylon* for their potential as biocontrol agents against the ash dieback pathogen and to define the genetic mechanisms encoding phomopsidin biosynthesis.

Last but not least, the current study also revealed some interesting aspects for potential follow-up projects. For instance, the examination of *H. fuscum* (a species that has never been isolated from *Fraxinus*, but is actually associated with *Corylus* and other Betulaceae) in the antagonism assay, revealed the production of several hitherto unknown compounds whose production was significantly enhanced in the presence of *Hym. fraxineus*. This observation suggests that it will be worthwhile to further study the secondary metabolism of *Hypoxylon* species in other scenarios using the dual culture approach. The first step would be to scale-up the production of the unknown molecules, isolating enough for structure elucidation and biological studies. This should not be expected to be a trivial task, but it appears doable using the methodology that is presently available.

The production of known and yet unidentified azaphilones (i.e. a compound class that is normally found in high concentrations in the stromata of various Hypoxylaceae, but was rarely observed in their mycelial cultures) in *H. rubiginosum* and allies, is another interesting observation relating to the differential expression of biosynthetic genes encoding secondary metabolites. It should be rewarding to evaluate the regulation mechanisms that lead to the production of the pigments, aided by genomic and transcriptomic studies.

## Supplementary Material

XML Treatment for Hypoxylon guilanense

XML Treatment for Hypoxylon rubiginosum

XML Treatment for Hypoxylon sp. aff. rubiginosum

XML Treatment for Hypoxylon sp. aff. rubiginosum

## Supplementary materials

Supplementary material 1 Discovery of a new species of the *Hypoxylon rubiginosum* complex from Iran and antagonistic activities of *Hypoxylon* species towards the Ash Dieback pathogen, *Hymenoscyphus fraxineus*, in dual culture Data type Multimedia. https://binary.pensoft.net/file/403613This dataset is made available under the Open Database License (http://opendatacommons.org/licenses/odbl/1.0/). The Open Database License (ODbL) is a license agreement intended to allow users to freely share, modify, and use this Dataset while maintaining this same freedom for others, provided that the original source and author(s) are credited.Mohammad Javad Pourmoghaddam, Christopher Lambert, Frank Surup, Seyed Akbar Khodaparast, Irmgard Krisai-Greilhuber, Hermann Voglmayr, Marc Stadler
