# Supplementary material for: Discovery of a new species of the Hypoxylon rubiginosum complex from Iran and antagonistic activities of Hypoxylon spp. against the Ash Dieback pathogen, Hymenoscyphus fraxineus, in dual culture
Source: MycoKeys. 2020 Apr 24;66:105–33. doi: 10.3897/mycokeys.66.50946 (PMC7195382; doi:10.3897/mycokeys.66.50946)

## Slide 1
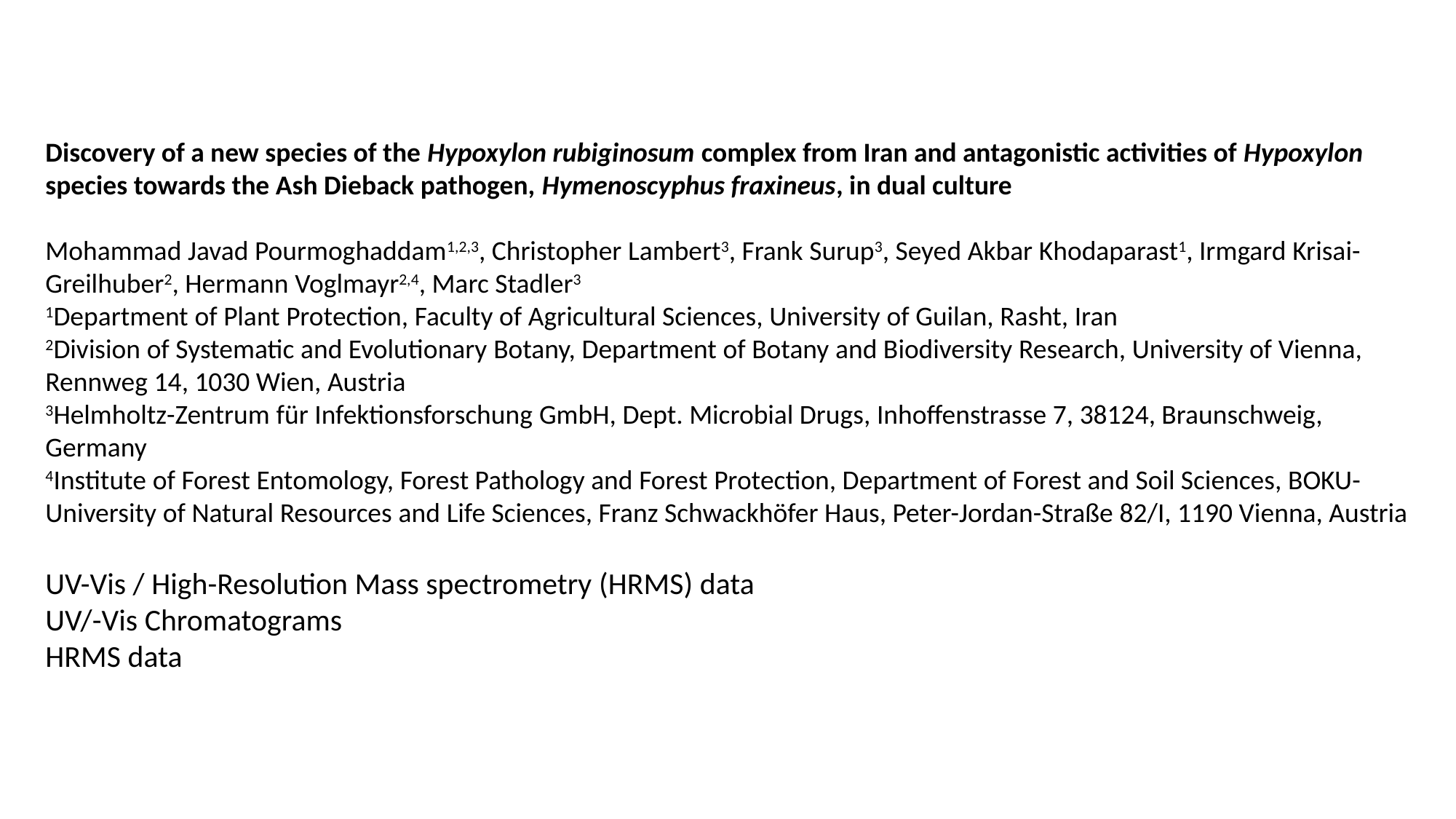

Discovery of a new species of the Hypoxylon rubiginosum complex from Iran and antagonistic activities of Hypoxylon species towards the Ash Dieback pathogen, Hymenoscyphus fraxineus, in dual culture
Mohammad Javad Pourmoghaddam1,2,3, Christopher Lambert3, Frank Surup3, Seyed Akbar Khodaparast1, Irmgard Krisai-Greilhuber2, Hermann Voglmayr2,4, Marc Stadler3
1Department of Plant Protection, Faculty of Agricultural Sciences, University of Guilan, Rasht, Iran
2Division of Systematic and Evolutionary Botany, Department of Botany and Biodiversity Research, University of Vienna, Rennweg 14, 1030 Wien, Austria
3Helmholtz-Zentrum für Infektionsforschung GmbH, Dept. Microbial Drugs, Inhoffenstrasse 7, 38124, Braunschweig, Germany
4Institute of Forest Entomology, Forest Pathology and Forest Protection, Department of Forest and Soil Sciences, BOKU-University of Natural Resources and Life Sciences, Franz Schwackhöfer Haus, Peter-Jordan-Straße 82/I, 1190 Vienna, Austria
UV-Vis / High-Resolution Mass spectrometry (HRMS) data
UV/-Vis Chromatograms
HRMS data

## Slide 2
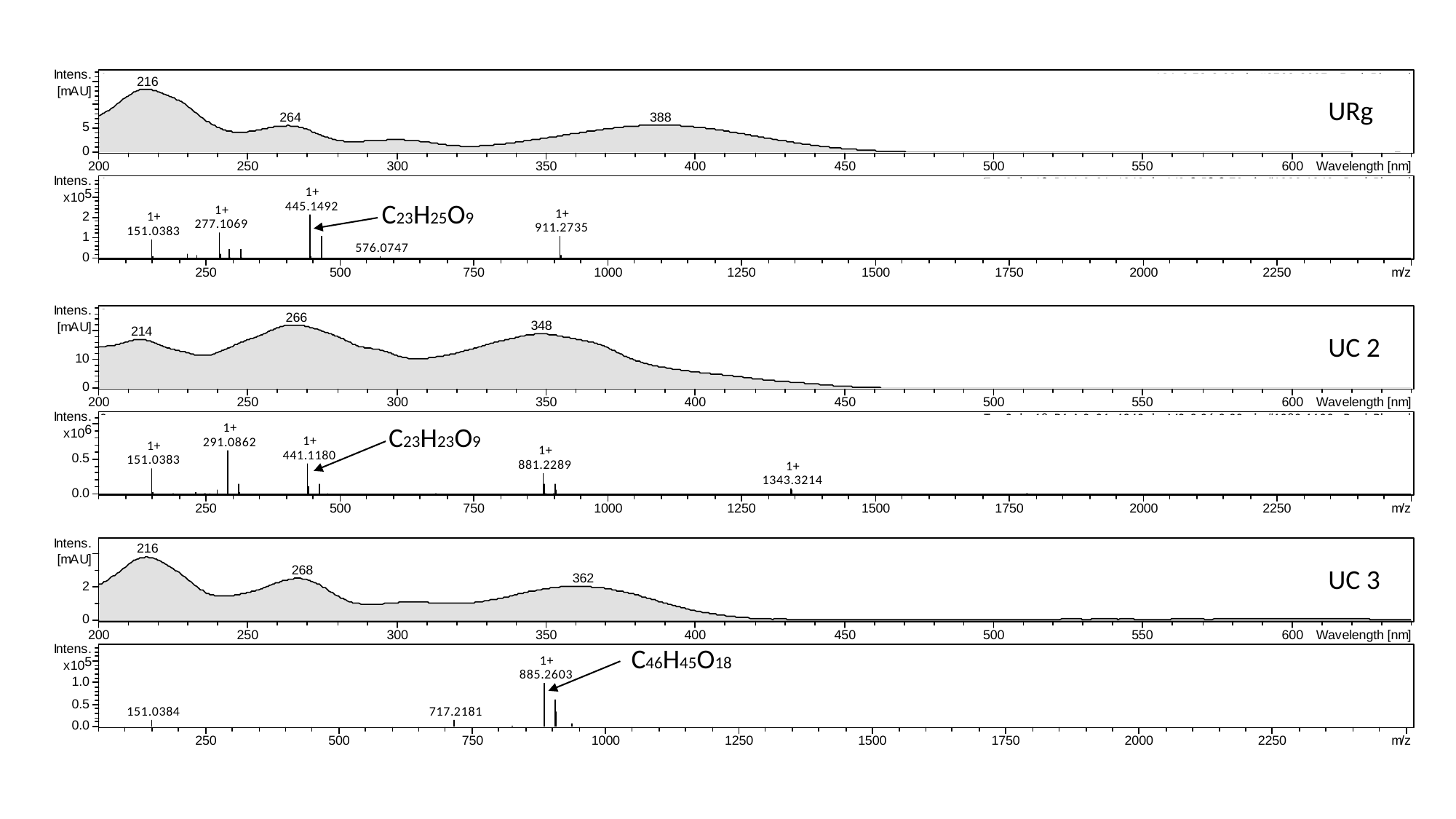

## Slide 3
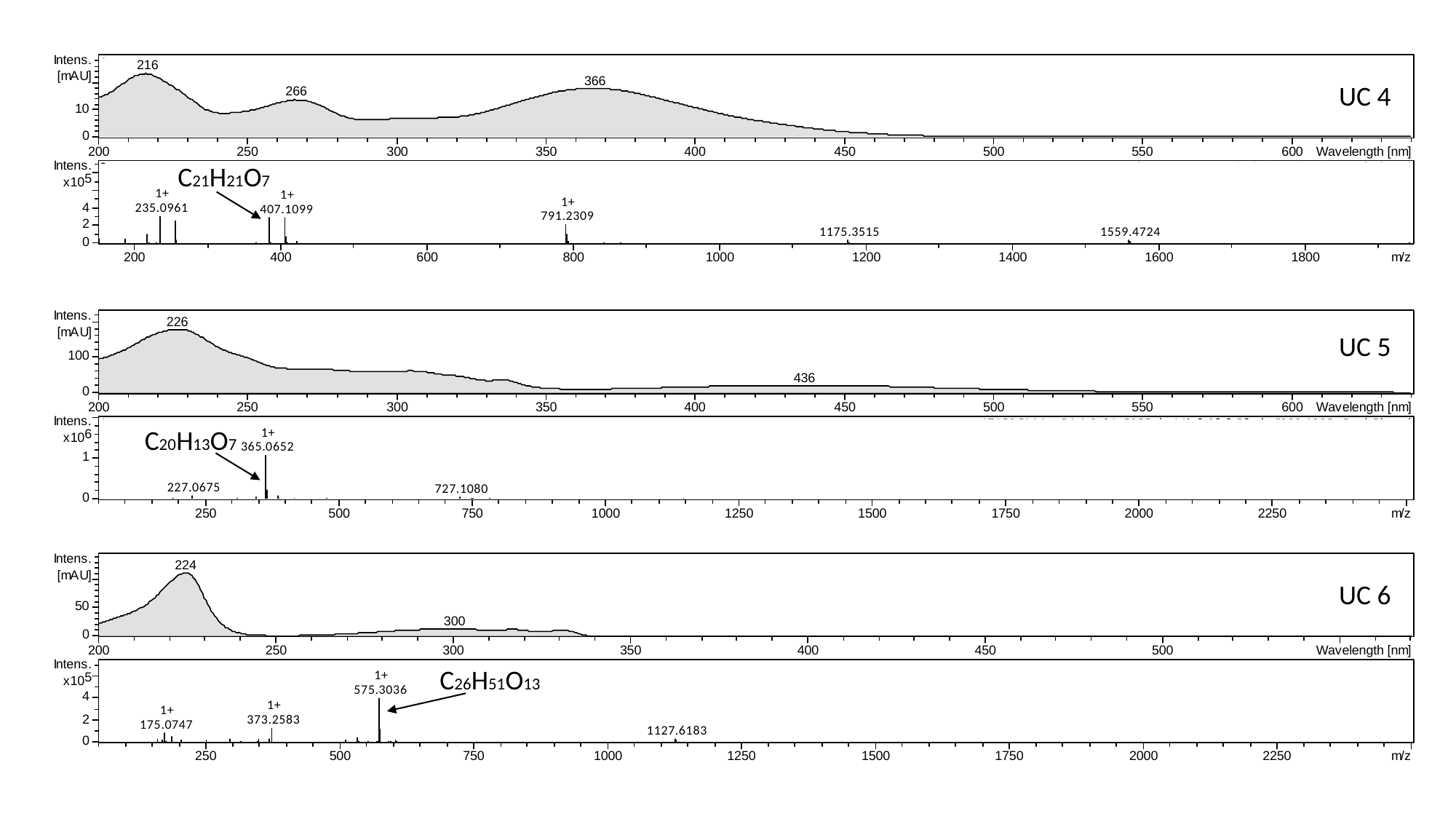

Supplement: Supplementary material 1 — Discovery of a new species of the Hypoxylon rubiginosum complex from Iran and antagonistic activities of Hypoxylon species towards the Ash Dieback pathogen, Hymenoscyphus fraxineus, in dual culture [file mycokeys-66-105-s001.pptx]
